# Multifunctional Nanofibrous Hollow Microspheres for Enhanced Periodontal Bone Regeneration

**DOI:** 10.1002/advs.202402335

**Published:** 2024-05-17

**Authors:** Qian Li, Chi Ma, Yan Jing, Xiaohua Liu

**Affiliations:** ^1^ Department of Biomedical Sciences Texas A&M University School of Dentistry Dallas TX 75246 USA; ^2^ Chemical and Biomedical Engineering Department University of Missouri Columbia MO 65211 USA; ^3^ Center of Excellence in Hip Scottish Rite for Children Dallas TX 75219 USA; ^4^ Department of Orthopedic Surgery University of Texas Southwestern Medical Center Dallas TX 75390 USA; ^5^ Department of Orthodontics Texas A&M University School of Dentistry Dallas TX 75246 USA

**Keywords:** alveolar bone regeneration, injectable, microspheres, nanofibers, periodontitis

## Abstract

Destructive periodontitis destroys alveolar bone and eventually leads to tooth loss. While guided bone regeneration, which is based on creating a physical barrier to hinder the infiltration of epithelial and connective tissues into defect sites, has been widely used for alveolar bone regeneration, its outcomes remain variable. In this work, a multifunctional nanofibrous hollow microsphere (NFHMS) is developed for enhanced alveolar bone regeneration. The NFHMS is first prepared via combining a double emulsification and a thermally induced phase separation process. Next, E7, a short peptide with high specific affinity to bone marrow‐derived stem cells (BMSCs), is conjugated onto the surface of NFHMS. After that, bone forming peptide (BFP), a short peptide derived from bone morphology protein 7 is loaded in calcium phosphate (CaP) nanoparticles, which are further encapsulated in the hollow space of the NFHMS‐E7 to form NFHMS‐E7‐CaP/BFP. The NFHMS‐E7‐CaP/BFP selectively promoted the adhesion of BMSCs and expelled the adhesion of fibroblasts and epithelial cells. In addition, the BFP is sustainedly released from the NFHMS‐E7‐CaP/BFP to enhance the osteogenesis of BMSCs. A rat challenging fenestration defect model showed that the NFHMS‐E7‐CaP/BFP significantly enhanced alveolar bone tissue regeneration. This work provides a novel bioengineering approach for guided bone regeneration.

## Graphical abstract

A multifunctional nanofibrous hollow microspheres (NFHMS) is developed as an injectable biomaterial for enhanced alveolar bone regeneration. The NFHMS acts as a biological barrier to selectively enrich BMSCs and repel the invasion of gingival fibroblasts and gingival epithelial cells. In addition, the sustained release of osteogenic factor significantly promotes alveolar bone regeneration.

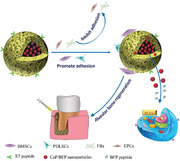

## 1 Introduction

Periodontitis is one of the most prevalent diseases, and it was estimated that 42% of U.S. adults aged 30 years or older have periodontitis.^[^ ^1^ ^]^ Destructive periodontitis destroys alveolar bone that houses and supports teeth, and eventually leads to tooth loss. Conventional therapies for periodontitis treatments, such as scaling and root planing, and flap surgery, can alleviate or halt the progression of the disease, but they cannot solve the problem of periodontal tissue loss, which undermines the long‐term efficacy of the treatments.^[^ ^2^ ^]^ To reconstruct the lost periodontal tissues and restore their functions, periodontal tissue regeneration has been extensively explored in recent years.^[^ ^3^ ^]^ Specifically, guided tissue/bone regeneration (GTR/GBR) has been clinically used for the treatment of alveolar bone loss.^[^ ^3^, ^4^ ^]^ GTR/GBR is a surgical procedure that uses resorbable or non‐resorbable membranes as physical barriers to prevent the migration of gingival fibroblasts (FBs) and gingival epithelial cells (EPCs) into the bony defect, thus providing a protective environment for bone marrow derived stem cells (BMSCs) migration, proliferation, differentiation, and formation of new alveolar bones. While GTR/GBR membranes can successfully inhibit the invasion of undesired FBs and EPCs, there are various limitations of these GTR/GBR physical barrier materials, including the inferior strength and unpredictable results for resorbable membranes and the high risk of exposure and infection as well as the necessity for a second surgery for non‐resorbable membranes.^[^ ^4^, ^5^ ^]^ Therefore, there is an unmet demand to develop new bioengineering strategies for periodontal bone regeneration.

E7 peptide is a novel peptide with the amino acid sequence of “EPLQLKM” identified through phage display technology, which has high specific affinity to BMSCs.^[^ ^6^ ^]^ E7 was recently applied to specifically enhance the adhesion and proliferation of BMSCs.^[^ ^7^ ^]^ In addition, an E7‐modified collagen was found to selectively capture BMSCs over fibroblasts and immune cells.^[^ ^8^ ^]^ Those reports suggest that the E7 peptide is likely to function as a biological barrier to selectively enrich BMSCs while excluding FBs and EPCs. To date, there has been no report on the development of biomaterials acting as a biological barrier to selectively enrich BMSCs while repel the invasion of FBs and EPCs.

Since periodontal bone resorption generally forms irregular alveolar bone defects, injectable biomaterials that can easily fill any shape of defects are more attractive than conventional pre‐formed 3D biomaterials.^[^ ^9^ ^]^ Hydrogels are injectable biomaterials and have been tested for bone formation.^[^ ^10^ ^]^ However, the limitations of hydrogels include the inferior mechanical property and poor integration with surrounding tissues. Besides hydrogels, microspheres with the diameters ranging from tens of micrometers to a few hundred micrometers are another type of injectable biomaterials that act as both cell carriers and drug delivery vehicles and have received increasing attentions in recent years.^[^ ^11^ ^]^ In contrast to hydrogels that usually do not allow cell migration until the outmost of the hydrogels have been dissolved or degraded, microspheres provide interspaces to facilitate cell migration and proliferation prior to the degradation of the microspheres, which can significantly accelerate new tissue regeneration. Conventional microspheres, however, have solid‐walled morphology and do not possess appropriate biophysical and/or biochemical cues to regulate cell‐material interactions to guide tissue regeneration. To fulfil the complicated requirements of periodontal tissue regeneration, functional microspheres that not only mimic the nanofibrous architecture of natural extracellular matrix (ECM), but also present desired biophysical and biochemical cues are expected.^[^ ^11^ ^]^

Growth factors play a pivotal role in tissue regeneration. Bone morphogenetic proteins (BMPs), such as BMP2 and BMP7, are potent osteo‐inductive growth factors and have been widely used to regulate new bone formation.^[^ ^12^ ^]^ However, BMPs are sensitive to physiological environment and are easily denatured. Short peptides are resistant to pH and thermal changes and can effectively circumvent the limitation of protein instability. Recently, a bone forming peptide (BFP) that is derived from a prodomain region of BMP7 was reported to activate osteogenesis.^[^ ^13^ ^]^ Because BFP is a 15 amino acid short peptide, it is not only structurally stable and resistant to denaturation, but also free from immunogenic issues.^[^ ^14^ ^]^ The BFP‐pretreated BMSCs had more bone formation in vivo compared to the BMP7‐pretreated BMSCs.^[^ ^13d^ ^]^ Therefore, BFP seems to be a promising candidate for accelerated alveolar bone regeneration.

**Scheme 1:**
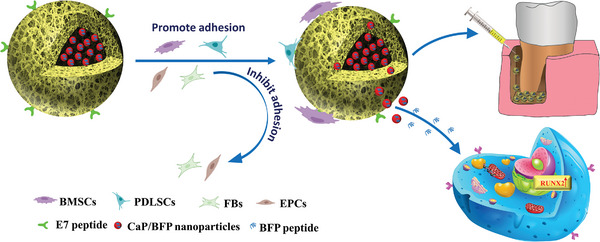
Schematic illustration of fabricating multifunctional NFHMS‐E7‐CaP/BFP microspheres for periodontal alveolar bone regeneration.

In this work, we report the development of multifunctional nanofibrous hollow microspheres (NFHMS) that possess three unique features to promote alveolar bone regeneration (**Scheme** **1**). First, the NFHMS were entirely composed of gelatin nanofibers, which mimic the composition and architecture of collagen in natural bone ECM and are expected to provide a favorable microenvironment for the migration, proliferation, and differentiation of BMSCs.^[^ ^15^ ^]^ Second, E7 peptide was conjugated onto the surface of the NFHMS to selectively enrich BMSCs and repel FBs and EPCs, functioning as a biological GTR/GBR membrane. Third, BFP peptide was loaded in calcium phosphate (CaP) nanoparticles which were further encapsulated into the hollow structure of the NFHMS. The BFP peptide, therefore, was released from the NFHMS in a precisely controlled manner to accelerate alveolar bone regeneration. We hypothesized that the hierarchical injectable NFHMS‐E7‐CaP/BFP would facilitate BMSC adhesion, proliferation, and differentiation, leading to enhanced alveolar bone regeneration.

## 2 Results

### 2.1 Synthesis and Characterization of Nanofibrous Hollow Microspheres (NFHMS)

**Figure 1:**
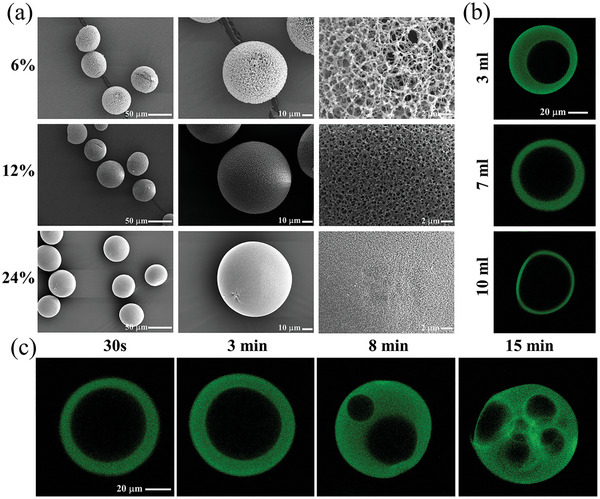
The morphologies of NFHMS in different parameters. a) gelatin concentration, b) volume of inner oil, and c) stirring time.

NFHMS were fabricated via a unique nanotechnology that integrates an O/W/O double emulsification with a thermally induced phase separation process (**Figure** **1**). Specifically, the O/W/O double emulsification was applied to generate hollow‐structured microspheres, while the thermally induce phase separation process was used to assemble gelatin macromolecular chains into nanofibers. As shown in Figure 1a, the NFHMS was entirely composed of nanofibers, mimicking the nanofibrous architecture of natural collagen fibers in bone ECM. A hollow structure with the pore size up to dozens of micrometers were generated inside the nanofibrous shell (Figure 1b,c).

The morphology and structure of NFHMS were readily adjusted via several fabrication parameters, including gelatin concentration, volume of inner oil, and stirring time (Table S2, Supporting Information). As the concentration of gelatin increased from 6% to 24%, the apparent density of NFHMS increased from 43.34 to 165.23 g dm^−3^, while the porosity decreased from 95.86% to 84.18%, respectively. Meanwhile, the average fiber diameter increased from 250 to 480 nm, and the average fiber length decreased from 1749 to 462 nm, respectively (Table S2, Supporting Information). The NFHMS had a much lower apparent density than that of the NFMS and solid MS. For example, the NFHMS fabricated with 12% gelatin had an apparent density of 76.01 g dm^−3^, which was less than 1/2 of that of the NFMS, and less than 1/14 of that of the solid MS (Table S2, Supporting Information). Lower apparent density of the NFHMS would generate significantly less degradation by‐products than that of the NFMS and solid MS. The volume of inner oil controlled the thickness of spherical shell, and the thickness decreased from 18.9±1.4 µm to 2.6±0.4 µm as the inner oil volume increased from 3 to 10 mL (Table S3, Supporting Information). Interestingly, the stirring time significantly influenced hollow structure formation. A single hollow pore was generated with short stirring time (≤ 3 min), while multiple hollow pores were generated with longer stirring time (Figure 1c). Due to the nanofibrous hollow structure, the NFHMS had a faster degradation rate than the NFMS and solid MS (Figure S1a, Supporting Information). After incubated in PBS for 35 days, 90.4% of the NFHMS were degraded, compared to 61.4% for the NFMS and 21.8% for the solid MS. Artificial saliva solution was further used to test the degradation of NFHMS, NFMS, and solid MS in a simulated oral microenvironment. The results showed similar degradation tendency compared with that incubated in PBS solution (Figure S1b, Supporting Information). Moreover, the average elastic modulus of NFHMS was 1.58 GPa, and the average hardness was 0.08 GPa (Figure S2, Supporting Information). In this work, the NFHMS fabricated under the conditions of 12% gelatin, 7 mL inner mineral oil, and 30s stirring time were selected for the rest the experiments.

### 2.2 Synthesis and Characterization of E7‐Modified CaP/BFP‐Loaded NFHMSs (NFHMS‐E7‐CaP/BFP)

**Figure 2:**
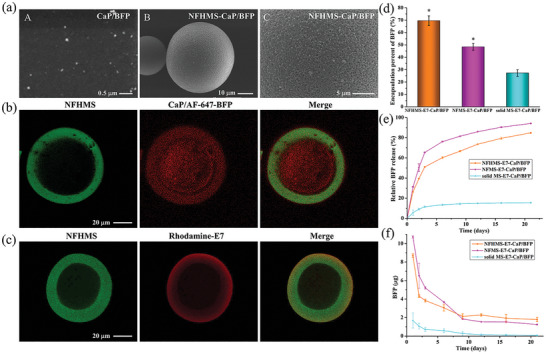
Characterizations of CaP/BFP‐loaded NFHMS and the release profiles. a) SEM images of NFHMS‐CaP/BFP and CaP/BFP nanoparticles. b) Cross‐sectional confocal images, showing the CaP/BFP NPs (red) were mainly distributed in the hollow area of the NFHMS (green). c) Confocal microscopy image of a NFHMS‐E7. d) The encapsulation percent of BFP in NFHMS‐E7‐CaP/BFP, NFMS‐E7‐CaP/BFP and solid MS‐E7‐CaP/BFP. e,f) Release profiles of BFP from NFHMS‐E7‐CaP/BFP, NFMS‐E7‐CaP/BFP and solid MS‐E7‐CaP/BFP. *N* = 3, *P < 0.05 versus NFHMS.

BFP‐loaded CaP NPs were synthesized using a precipitation process. The average size of the nanoparticles was 103±15 nm (**Figure** **2**a). The CaP/BFP NPs were encapsulated into NFHMS during the process of thermally induced phase separation. The appearance of the peak at the wavenumber of 1117 cm^−1^ that is assigned to (HPO_4_)^2−^ indicated the successful encapsulation of CaP/BFP NPs into the NFHMS (Figure S3, Supporting Information). The energy dispersive X‐ray spectroscopy (EDS) further indicated the distributions of the phosphorus and calcium elements on the NFHMS, confirming the encapsulation of the CaP/BFP NPs into the NFHMS (Figure S4, Supporting Information). The incorporation of CaP/BFP into NFHMS did not affect the formation of gelatin nanofibers. However, the nanofibers on the surfaces of the NFHMS‐CaP/BFP were denser compared to the NFHMS (Figure 2a, Figure 1b). The sizes of the NFHMS‐CaP/BFP ranged from 32–150 µm, and most of them were at the range of 45–90 µm under the experimental condition (Figure S5, Supporting Information).

To visualize the distribution of CaP/BFP in the NFHMS‐CaP/BFP, fluorescein isothiocyanate (FITC)‐conjugated gelatin and rhodamine‐conjugated BFP were used to fabricate NFHMS‐CaP/BFP. As shown in Figure 2b, most of the CaP/BFP NPs (red) were entrapped in the hollow core of the NFHMS (green), while a small number of the CaP/BFP NPs were entangled in the nanofibrous shell of the NFHMS. Similarly, gelatin was labeled with FITC and E7 was labeled with rhodamine to visualize the conjugation of E7 on the surface of NFHMS. As shown in Figure 2c, a bright red ring encircled the NFHMS (green), indicating E7 was evenly distributed on the surface of the NFHMS. To quantitatively measure E7 peptide content in NFHMS‐E7, tryptophan residue (W), which shows specific fluorescence emission spectrum at 350 nm, was combined with the E7 sequence. As shown in Figure S6, the E7 peptide content was 3.0% in NFHMS‐E7. The standard curve is *Y*  = 603416.43$\;\times \;C(\frac{mg}{mL})-3261.79.$

The encapsulation efficiency of CaP/BFP NPs in NFHMS‐E7‐CaP/BFP, NFMS‐E7‐CaP/BFP and solid MS‐E7‐CaP/BFP was 69.5±3.8%, 48.4±2.7% and 27.2±2.7%, respectively (Figure 2d). The high encapsulation efficiency of NFHMS‐E7‐CaP/BFP was ascribable to the hollow structure of NFHMS.

BSA was first used as a model protein to test the release profile of proteins from the CaP NPs and NFHMS‐CaP (Figure S7, Supporting Information). At day 6, almost all the BSA was released from the CaP/BSA NPs. In contrast, there was still 37.3% of the BSA remained in the NFHMS‐CaP/BSA. This result indicated that encapsulation of CaP/BSA NPs into NFHMS provided a better sustained release of proteins from the microspheres. Next, the CaP/BFP was encapsulated into NFHMS‐E7, NFMS‐E7, and solid MS‐E7 to examine the release of BFP from the microspheres (Figure 2e,f). The release of BFP from the NFHMS‐E7‐CaP/BFP, NFMS‐E7‐CaP/BFP and solid MS‐E7‐CaP/BFP on the first day was 26.3±0.6%, 31.2±0.4%, and 5.6±2.7%, respectively. While the MS‐E7‐CaP/BFP had the lowest burst release, the overall release rate of BFP from the solid MS was slow. In fact, only approximately 15.5% of BFP was released from the solid MS after 21 days. In contrast, the BFP in the NFHMS‐E7‐CaP/BFP was sustainedly released for over three weeks, and over 84.9% of the BFP was released from the NFHMS‐E7‐CaP/BFP by day 21.

### 2.3 Selective Cell Adhesion on NFHMS and NFHMS‐E7

**Figure 3:**
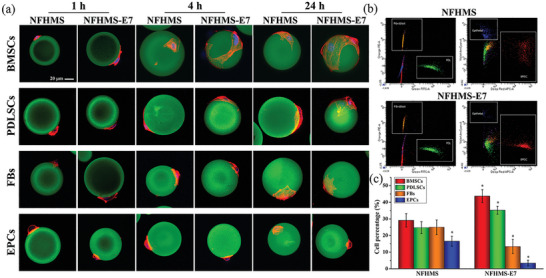
a) Confocal microscopy images of BMSCs, PDLSCs, FBs, and EPCs attached on the surfaces of NFHMS and NFHMS‐E7 at different time points after seeding. b,c) Cell percentage of BMSCs, PDLSCs, FBs, and EPCs measured by flow cytometry. *N* = 3, *P < 0.05.

To examine the effect of E7 peptide on cell adhesion, bone marrow derived stem cells (BMSCs), Periodontal ligament cells (PDLSCs), Fibroblasts (FBs), and Epithelial cells (EPCs) were seeded, separately, on the NFHMS and NFHMS‐E7 for 1, 4, and 24 h. As shown in **Figure** **3**a, the four types of cells had different morphologies on the NFHMS and NFHMS‐E7. At the same time point, the spreading area of the BMSCs on the NFHMS‐E7 was significantly greater than that on the NFHMS. At 4 h, the BMSCs on the NFHMS‐E7 spread to exhibit well‐arranged cytoskeleton. At 24 h, the BMSCs were fully spreading and covered more surface area of the NFHMS‐E7. Conversely, the spreading rate of the BMSCs on the NFHMS was substantially slower, and the spreading area of the BMSCs on the NFHMS at 24 h was smaller than that on the NFHMS‐E7 at 4 h, indicating that E7 significantly promoted BMSCs adhesion on the NFHMS‐E7. Compared to the BMSCs, the FBs and EPCs spread much more slowly on the NFHMS‐E7. At 24 h, the FBs on the NFHMS‐E7 were partially spreading, while the EPCs remained a rounded morphology on the NFHMS‐E7.

To further investigate the competitive adhesion of the four types of the cells onto the NFHMS‐E7, BMSCs, PDLSCs, FBs, and EPCs were mixed and seeded onto the microspheres. Each cell type with the same cell number was labeled with cell‐tracking dyes prior to seeding onto the NFHMS and NFHMS‐E7. At 24 h, the adherent cells were harvested for flow cytometry to analyze the ratio of each type of cells on the microspheres (Figure 3b). In the NFHMS group, the BMSCs and PDLSCs slightly increased the ratios in the total adherent cells, while the number of the EPCs was significantly decreased (Figure 3c). In the NFHMS‐E7 group, the competitive adhesion advantage of the BMSCs were clearly observed, the BMSCs in the NFHMS‐E7 were 43.7%, which was 1.5‐fold higher than that in the NFHMS. The number of FBs in the NFHMS‐E7 decreased by 11.6% compared to that in the NFHMS. For the EPCs, the adhesion ratio was reduced to 3.4% on the NFHMS‐E7 compared to 16.6% on the NFHMS. These results indicated that NFHMS‐E7 selectively enhanced the competitive adhesion of BMSCs and suppressed the competitive adhesion of FBs and EPCs.

### 2.4 BMSCs Proliferation, Differentiation, and Mineralization on NFHMS‐E7‐CaP/BFP

**Figure 4:**
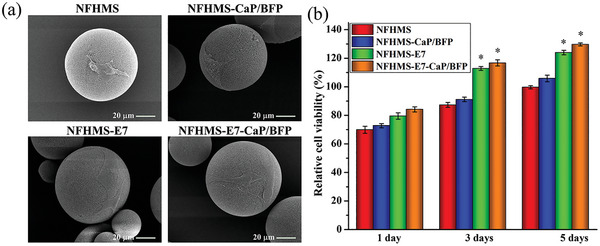
Cell adhesion and proliferation on the NFHMS, NFHMS‐CaP/BFP, NFHMS‐E7 and NFHMS‐E7‐CaP/BFP groups. a) SEM images of BMSCs attached on the surfaces of NFHMS, NFHMS‐CaP/BFP, NFHMS‐E7, and NFHMS‐E7‐CaP/BFP groups 24 h after cell seeding. b) Relative cell viability of the cells on the NFHMS, NFHMS‐CaP/BFP, NFHMS‐E7, and NFHMS‐E7‐CaP/BFP after cultured for 1, 3, and 5 days. *N* = 3, *P < 0.05 versus NFHMS.

To examine the effect of BFP on BMSCs, the BMSCs were seeded onto the NFHMS, NFHMS‐CaP/BFP, NFHMS‐E7, and NFHMS‐ E7‐CaP/BFP and cultured for up to 5 days. At 24 h after cell seeding, the BMSCs attached and spread well on the NFHMS, NFHMS‐CaP/BFP, NFHMS‐E7, and NFHMS‐E7‐CaP/BFP (**Figure** **4**a). An MTT assay was preformed to evaluate the effect of BFP concentration on BMSCs (Figure S8, Supporting Information). After incubation of 3 days, all the groups showed high cell viability (>99%) in a wide range of BFP concentrations (0.01 µg mL^−1^ ‐100 µg mL^−1^), suggesting that the concentration of BFP did not affect BMSCs viability. In addition, the BMSCs on all four groups proliferated with culture time, indicating the excellent biocompatibility of the NFHMS, NFHMS‐CaP/BFP, NFHMS‐E7, and NFHMS‐ E7‐CaP/BFP. Moreover, the cell numbers on the NFHMS‐E7 and NFHMS‐E7‐CaP/BFP groups were significantly higher than that on the NFHMS and NFHMS‐CaP/BFP on days 3 and 5, separately (Figure 4b). Meanwhile, the cell numbers had no statistical difference between the NFHMS‐E7 and NFHMS‐E7‐CaP/BFP, confirming that the conjugated E7 peptide on the surfaces of the NFHMS and NFHMS‐CaP/BFP promoted BMSCs attachment and proliferation.

To determine the differentiation effect of BFP, BMSCs were treated with various concentrations of BFP, and the expressions of osteogenic genes (ALP, RUNX2, SP7, and OCN) were examined 14 days after cell cultures (Figure S9, Supporting Information). Overall, the expression levels of ALP, RUNX2, SP7, and OCN increased with the concentration of BFP, and reached the highest values at the concentration of 1 µg mL^−1^. Further increasing the concentration of BFP led to a slight decrease of the expressions of these osteogenic genes. Based on the above result, BFP with a concentration of 3.5 µg BFP mg^−1^ NFHMS was encapsulated in the microspheres to evaluate its osteogenic effect on BMSCs differentiation and mineralization. The BMSCs were seeded on the NFHMS, NFHMS‐CaP/BFP, NFHMS‐E7, and NFHMS‐E7‐CaP/BFP and cultured in the osteogenic medium for 7 and 14 days prior to the examination of the differentiation and mineralization. Overall, the expression levels of the osteogenic markers (ALP, RUNX2, SP7, and OCN) in the NFHMS, NFHMS‐CaP/BFP, NFHMS‐E7, and NFHMS‐E7‐CaP/BFP increased with the culture time. In addition, the expression levels of the three early osteogenic markers (ALP, RUNX2, and SP7) in the NFHMS‐CaP/BFP and NFHMS‐E7‐CaP/BFP were significantly higher than those in the NFHMS and NFHMS‐E7 at day 7. The OCN had a low expression among all four groups at day 7, probably owing to the late osteogenic marker of OCN. At day 14, the expression levels of ALP, RUNX2, SP7, and OCN in the NFHMS‐E7‐CaP/BFP group were 7.7‐, 7.9‐, 12.3‐, and 9.8‐fold higher than those in the NFHMS groups, and 1.2‐, 1.4‐, 1.6‐, and 1.9‐fold higher than those in the NFHMS‐CaP/BFP group, respectively. These results indicated that incorporation of BFP into the NFHMS significantly promoted BMSCs differentiation.

**Figure 5:**
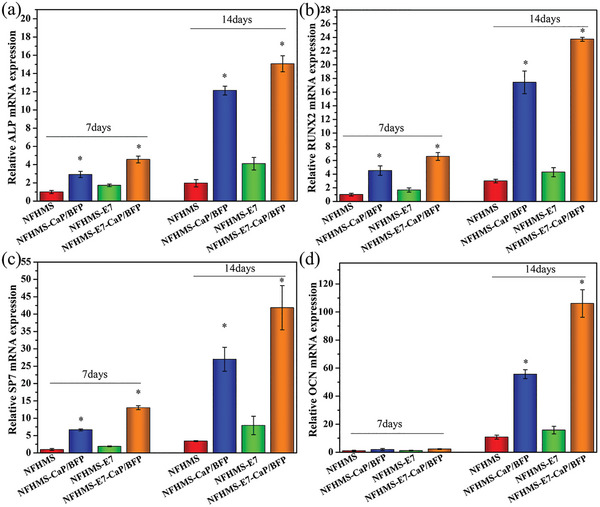
Osteogenic differentiation of BMSCs on the NFHMS, NFHMS‐CaP/BFP, NFHMS‐E7, and NFHMS‐E7‐CaP/BFP after the cells were cultured on microspheres for 7 and 14 days. a) ALP expression, b) RUNX2 expression, c) SP7 expression, and d) OCN expression. *N* = 3, *P < 0.05 versus NFHMS.

**Figure 6:**
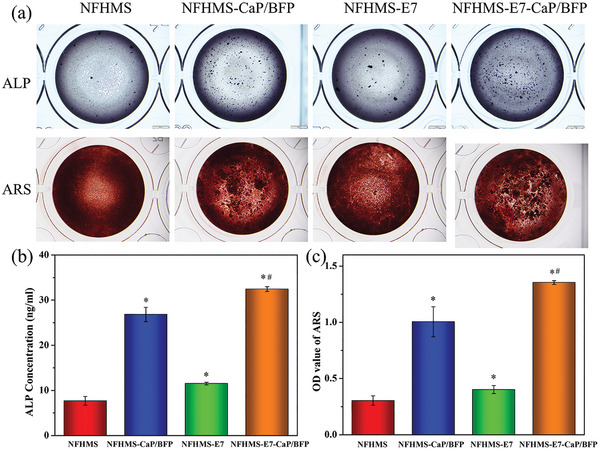
a) ALP and ARS staining after cells were cultured on the NFHMS, NFHMS‐CaP/BFP, NFHMS‐E7, and NFHMS‐E7‐CaP/BFP for 7 and 14 days, separately. b) Quantitative analyses of the ALP expression in different groups. c) Quantitative analyses of the calcium deposition in different groups. *N* = 3, *P < 0.05 versus NFHMS, ^#^ *P* < 0.05 versus NFHMS‐E7.

ALP staining and ARS staining were further performed to evaluate BMSCs differentiation and mineralization on the NFHMS, NFHMS‐CaP/BFP, NFHMS‐E7 and NFHMS‐E7‐CaP/BFP (**Figure** **5**). At day 7, the ALP concentration in the NFHMS‐E7 group was 1.5‐fold higher than that in the NFHMS. Subsequently, the ALP concentration in the NFHMS‐E7‐CaP/BFP group was 4.2‐, 1.2‐, and 2.8‐fold higher than that in the NFHMS, NFHMS‐CaP/BFP and NFHMS‐E7, respectively (**Figure** **6**b). At day 14, ARS staining revealed more calcium deposition in the NFHMS‐E7‐CaP/BFP compared with other groups. The relative calcium concentration in the NFHMS‐E7‐CaP/BFP was 4.5‐, 1.3‐, and 3.4‐fold higher than that of the NFHMS, NFHMS‐CaP/BFP and NFHMS‐E7, respectively (Figure 6c). Collectively, these data suggested that the introduction of CaP/BFP NPs in the NFHMS‐E7‐CaP/BFP significantly enhanced osteogenic differentiation and mineralization.

### 2.5 In Vivo Periodontal Alveolar Bone Regeneration

**Figure 7:**
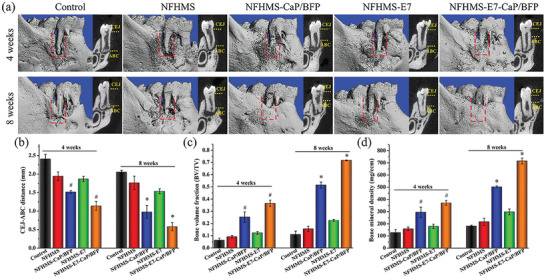
a) μ‐CT images of defects area and regenerated bone at 4 weeks and 8 weeks after implantation. b) Distance between the CEJ and ABC of the first molar distal root. c) Quantitative analysis of BV/TV. d) Bone mineral density (BMD) values of different groups. *N* = 4, ^#^ *P*< 0.05, *P < 0.05.

A rat mandibular fenestration defect that connects the defect to the oral cavity was created to examine the effect of NFHMS‐E7‐CaP/BFP on in vivo bone regeneration. A total of 40 SD rats were randomly assigned to 5 groups: control, NFHMS, NFHMS‐CaP/BFP, NFHMS‐E7, and NFHMS‐E7‐CaP/BFP. The animals were sacrificed at 4 and 8 weeks after the surgery. The µ‐CT images showed that a small amount of mineralized tissue formed in the defect area of the control group at 4 weeks after implantation (**Figure** **7**a). Some new bone appeared in the defect of the NFHMS and NFHMS‐E7 treated groups. However, significantly more newly formed bone covered the defect area in the NFHMS‐CaP/BFP and NFHMS‐E7‐CaP/BFP. The distance of CEJ to ABC in the distal area was measured to observe the impact of BFP and E7 on periodontal bone regeneration (Figure 7b). At 4 weeks, the distance from CEJ to ABC in the control group was longer than that in the NFHMS and NFHMS‐E7 groups, while the distance in the NFHMS‐CaP/BFP and NFHMS‐E7‐CaP/BFP was shorter than that in the NFHMS and NFHMS‐E7, separately. Eight weeks after implantation, the distance from CEJ to ABC in the NFHMS‐E7‐CaP/BFP group was the shortest among all five groups. In addition, the areas treated with NFHMS‐E7‐CaP/BFP had the highest BV/TV values at both time points (Figure 7c). Specifically, the value of the BV/TV in the NFHMS‐E7‐CaP/BFP at 8 weeks was 6.5‐, 4.6‐, 1.4‐, and 3.2‐fold higher than that in the control, NFHMS, NFHMS‐CaP/BFP and NFHMS‐E7, respectively (Figure 7c). In addition, the regenerated bone mineral density (BMD) in the NFHMS‐E7‐CaP/BFP group at 8 weeks was 715.2 mg ccm^−1^, which was 3.9‐, 3.3‐, 1.4‐, and 2.4‐fold higher than that in the control, NFHMS, NFHMS‐CaP/BFP, and NFHMS‐E7 groups, respectively (Figure 7d).

**Figure 8:**
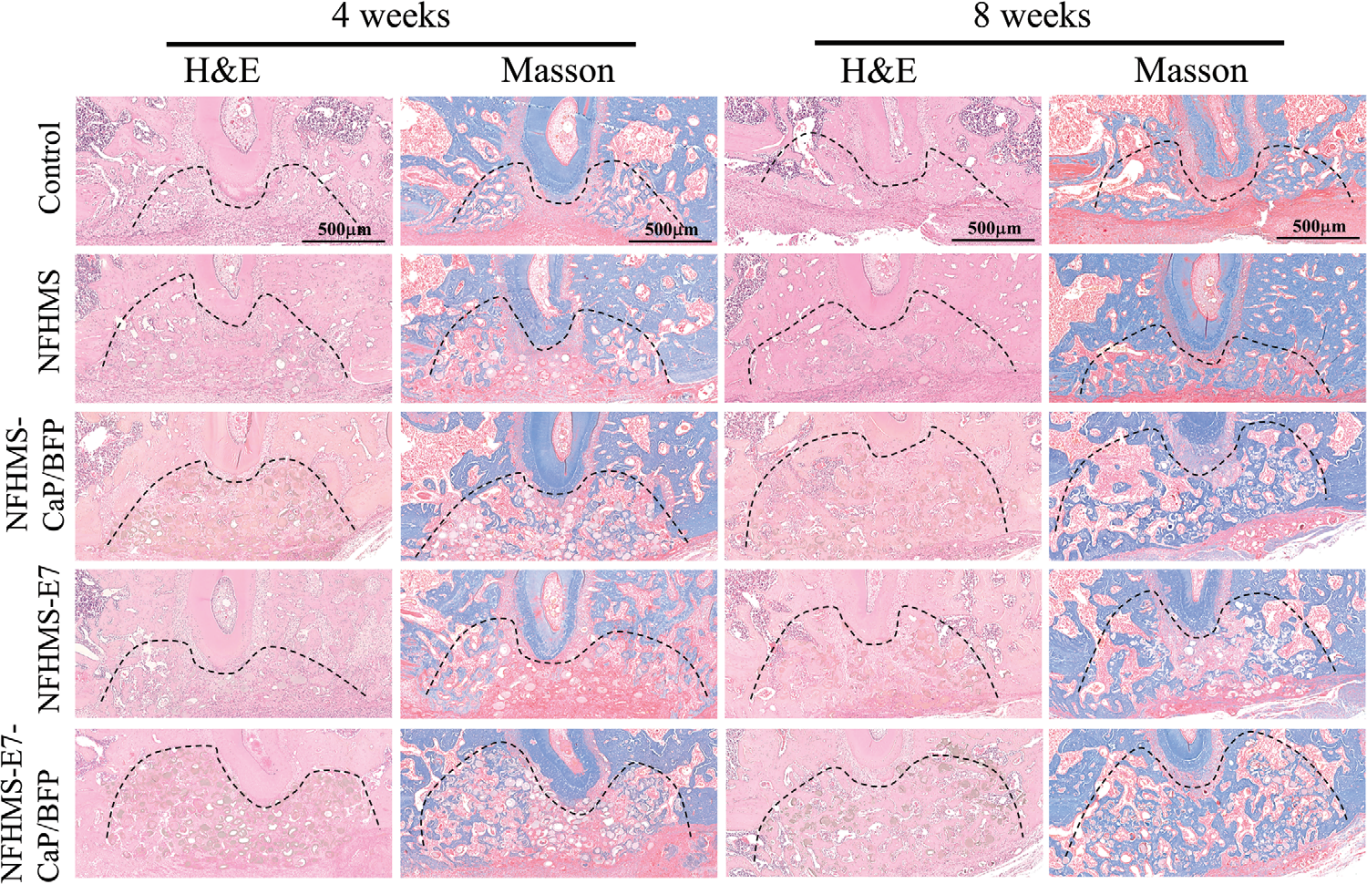
H&E staining and Masson's trichrome staining of different groups at 4 weeks and 8 weeks after implantation.

H&E staining and Masson's trichrome staining were performed to evaluate newly formed bone tissue (**Figure** **8**). At 4 weeks post‐surgery, the defect area of the control group showed scarce new bone in the tooth root side and the rest of the area was filled with fibrous tissue. A small amount of bone formation was observed in the NFHMS and NFHMS‐E7 groups. In contrast, in the NFHMS‐CaP/BFP and NFHMS‐E7‐CaP/BFP groups, more new bone occupied in the defect area. At 8 weeks after implantation, the new bone almost covered all the defect area in the NFHMS‐E7‐CaP/BFP group. In addition, the newly formed bone had a high density in the defect area compared to other four groups, which is consistent with the μ‐CT result (Figure 7a).

**Figure 9:**
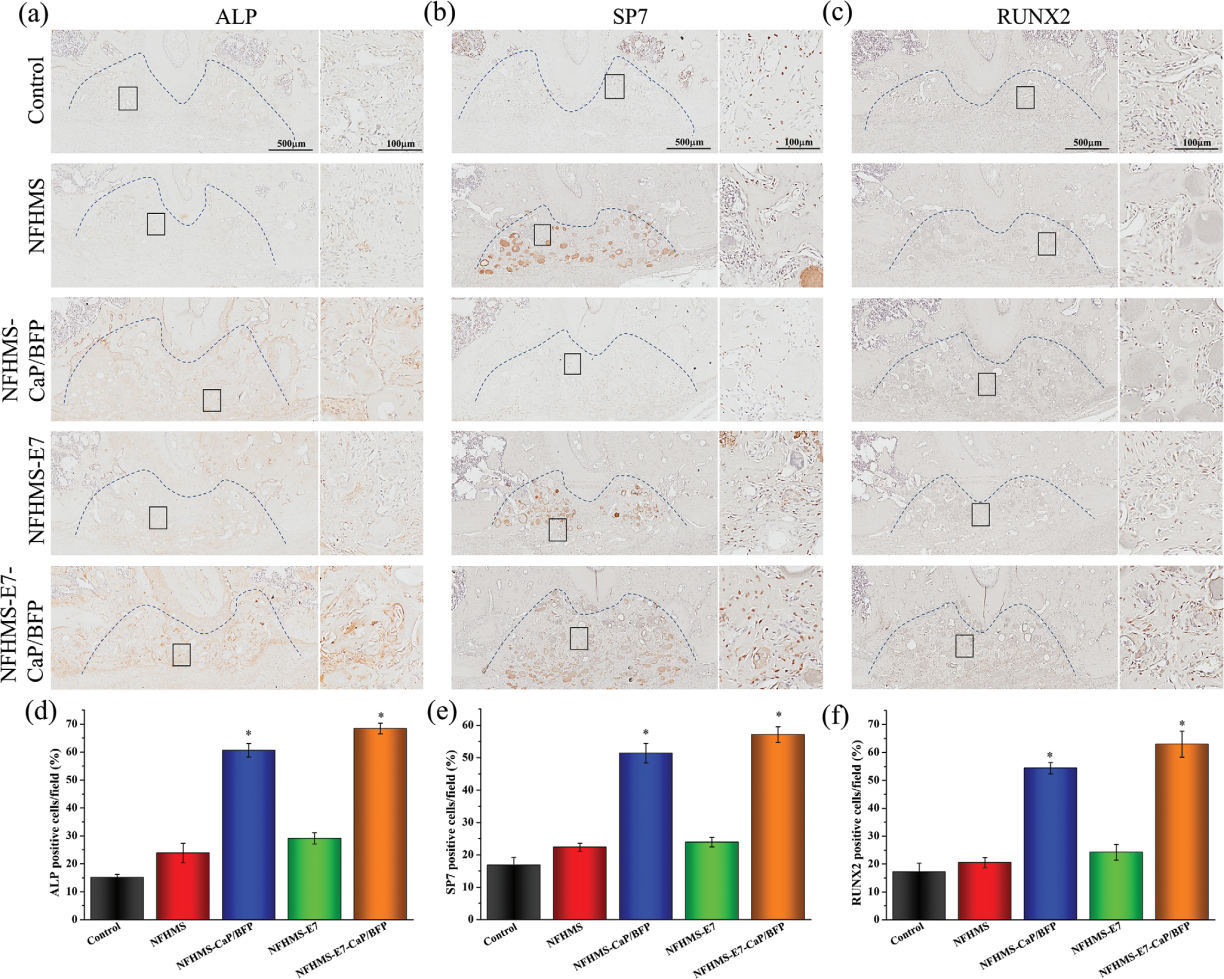
Immunohistochemical staining images of ALP a), SP7 b), and RUNX2 c) in the defects area 4 weeks after implantation. d–f) Quantitative analysis of the ratios of positive cells in the region of interest. d) ALP positive, e) SP7 positive, and f) RUNX2 positive. *N* = 4, *P < 0.05.

**Figure 10:**
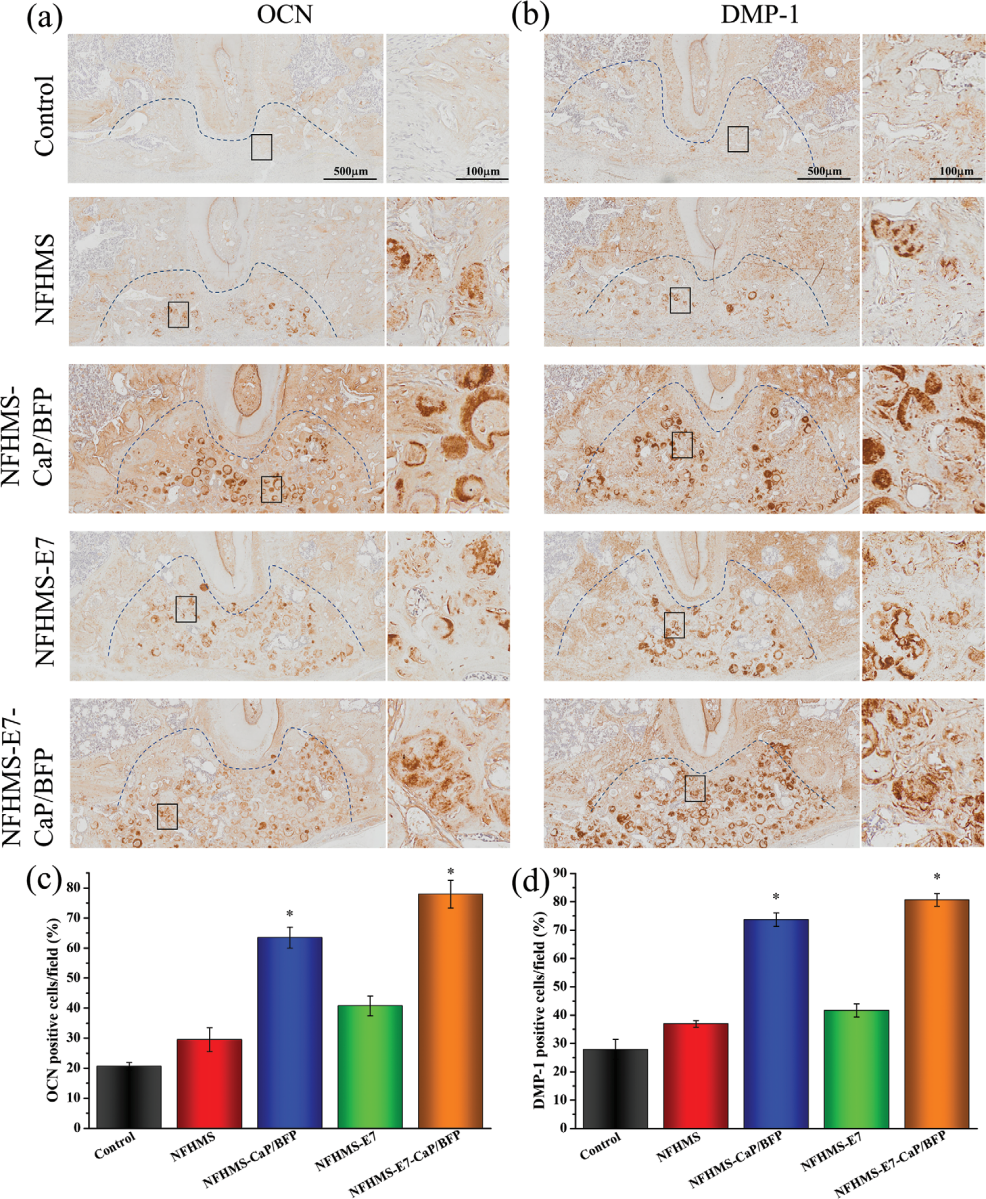
Immunohistochemical staining images of OCN a) and DMP‐1 b) in the defects area 8 weeks after implantation. (c‐d) Quantitative analysis of the ratios of positive cells in the region of interest. c) OCN positive, and d) DMP‐1 positive. *N* = 4, *P < 0.05.

IHC staining was used to further evaluate the osteogenesis of the control, NFHMS, NFHMS‐CaP/BFP, NFHMS‐E7, and NFHMS‐E7‐CaP/BFP. After implantation for 4 weeks, the early osteogenic markers (ALP, RUNX2 and SP7) in the NFHMS‐E7‐CaP/BFP group were much stronger than those in the control, NFHMS, and NFHMS‐E7 groups, and a little higher than those in the NFHMS‐CaP/BFP group (**Figure** **9**). Quantitative analysis indicated that the ratios of positive cells of ALP, RUNX2, and SP7 in the NFHMS‐E7‐CaP/BFP group were 4.5‐, 3.7‐, and 3.4‐fold higher than those in control group, 2.9‐, 3.1‐, and 2.6‐fold higher than those in NFHMS group, and 2.4‐, 2.6‐, and 2.4‐fold higher than those in NFHMS‐E7 group, respectively. By 8 weeks, the expressions of OCN and DMP‐1 (two late osteogenic markers) in the NFHMS‐E7‐CaP/BFP group were much stronger than those in the other four groups (**Figure** **10**). The positive cell ratios of OCN and DMP‐1 in the NFHMS‐E7‐CaP/BFP group were 3.8‐ and 2.9‐fold higher than those in the control group, 2.6‐, and 2.2‐fold higher than those in the NFHMS group, 1.9‐ and 1.9‐fold higher than those in the NFHMS‐E7 group, respectively. Taken together, the NFHMS‐E7‐CaP/BFP group presented the best result for enhanced alveolar bone regeneration.

## 3 Discussions

Periodontal bone regeneration typically involves various surgical and regenerative techniques to rebuild the lost alveolar bone and improve the overall health. One widely used approach is GTR/GBR that uses a membrane as a physical barrier to prevent the fast growth of soft gum tissues.^[^ ^16^ ^]^ The outcome of this technique, however, is often unpredictable for several periodontal defects (e.g., a fenestration defect that connects the defect to the oral cavity). In this work, we developed a new type of injectable biomaterial ─ multifunctional nanofibrous hollow microspheres ─ for alveolar bone regeneration. Our results show that the multifunctional nanofibrous hollow microspheres (NFHMS‐E7‐CaP/BFP) successfully repaired the alveolar bone defect in a challenging fenestration model, indicating that the NFHMS‐E7‐CaP/BFP is a promising biomaterial for periodontal bone regeneration.

One unique feature of the NFHMS is the nanofibrous hollow core/shell structure. While there were some reports on the fabrication of nanofibrous microspheres for tissue regeneration,^[^ ^17^ ^]^ the fabrication of nanofibrous hollow microspheres has long been a challenge. The advantage of incorporating hollow structure into nanofibrous microspheres is to facilitate the encapsulation of bioactive molecules and control of their release from the microspheres to precisely regulate tissue regeneration. In this work, we developed a technology that integrates a double emulsification and a thermally induced phase separation process to fabricate nanofibrous hollow gelatin microspheres. Several fabrication parameters can be used to readily control the architecture and properties of the NFHMS. For example, a low gelatin concentration formed microspheres with a low apparent density, a high porosity, and long gelatin nanofibers. A low apparent density of the microspheres generates small amount of degradation by‐products, and a high porosity provides more space for cell adhesion and growth. However, the NFHMS fabricated with a low gelatin concentration (e.g., 6%) were easily distorted. The size of the hollow structure was adjusted by the volume ratio of inner oil/gelatin solution during the fabrication process. Increasing the volume ratio of inner oil/gelatin solution resulted in the formation of a larger hollow structure and simultaneously a reduced thickness of the nanofibrous shell of microspheres. As the volume ratio of inner oil/gelatin solution increased to 7/10, the thickness of the nanofibrous shell of the NFHMS was 11.3±0.6 µm. Further increasing the volume ratio of inner oil/gelatin solution led to the NFHMS with susceptible breakage of the nanofibrous shell.

Interestingly, the stirring time was a crucial factor to control the formation of single or multiple hollow structures within the NFHMS. Only one hollow pore was formed with a short stirring time, while multiple hollow pores were formed with a prolonged stirring time (≥ 8 min). This is likely because the inner oil droplets were relatively large in the continuous gelatin solution phase with a short stirring time, while the inner oil droplets became smaller as the stirring time increased, which allowed the gelatin liquid beads to encapsulate more inner oil droplets. The detailed mechanism warrants further investigation. Given the objective of this work was to encapsulate a single growth factor (BFP) within each NFHMS, we chose the stirring time of 30 s to fabricate the NFHMS with a solitary hollow structure. The hollow and nanofibrous architecture of the NFHMS provided a high encapsulation efficiency (up to 69.5±3.8%) and more space for cell adhesion and growth.

Another distinctive design of this work is the incorporation of E7 peptide onto the nanofibrous surface of the NFHMS to selectively promote BMSCs adhesion and growth and prevent FBs and EPCs adhesion and migration. E7 peptide has a high affinity to BMSCs, therefore, the NFHMS‐E7 served as a biological barrier to inhibit FBs and EPCs migration during alveolar bone regeneration. The selective adhesion of BMSCs to the NFHMS‐E7 was verified through co‐culturing BMSCs, PDLSCs with FBs and EPCs onto the NFHMS‐E7. Unlike the GTR/GBR membranes that block all cells (including BMSCs) on the barrier side of the membrane, the NFHMS‐E7 shows selective affinity to the BMSCs in the vicinity of the periodontal defect. E7 peptide was acquired through an affinity selection technique called phage display biopanning to screen peptides that binds to BMSCs in a positive screening process and excludes peptides binding to fibroblasts in a negative screening process. In addition, the incorporation of the E7 peptide onto the NFHMS obscures the presentation of the innate Arg‐Gly‐Asp (RGD) sequence of gelatin on the surface of the NFHMS‐E7, resulting in fewer binding sites for GFs and reduced competitive adhesion compared to BMSCs. Meanwhile, EPCs adhere to ECM mainly through hemidesmosomes (HDs), and laminin is an indispensable component for the formation HDs. The structural difference between gelatin and laminin likely contributes to the low adhesion of EPCs on the NFHMS‐E7. In addition, the E7‐based biological barrier eliminates of the nutrient diffusion limitation of GTR/GBR membranes during tissue regeneration. Therefore, the NFHMS‐E7 provides an excellent microenvironment to recruit BMSCs and promote their growth in the defect area. Osteo‐inductive factors, especially BMPs, have been widely used to accelerate BMSCs differentiation and mineralization.^[^ ^18^ ^]^ However, BMPs are proteins and tend to lose bioactivities due to their fragile structural conformation. In addition, a growing and well‐documented side effect profile of BMPs clinical use has emerged.^[^ ^19^ ^]^ In this work, we selected BFP peptide that is a 15 amino acid short peptide derived from a prodomain region of BMP7. Due to its small molecular weight, BFP is not only structurally stable and resistant to denaturation, but also free from immunogenic issues.^[^ ^14^ ^]^ The BFP was reported to highly activate osteogenesis both in vitro and in vivo.^[^ ^13^ ^]^

To achieve well‐defined release of BFP from the NFHMS, we first encapsulated the BFP into CaP nanoparticles, which were further entrapped in the NFHMS. Therefore, the BFP was released in a dual‐controlled manner from the NFHMS. During the release process, the BFP in the NFHMS‐CaP/BFP needs to escape the entrapment of CaP NPs and overcome the physical adsorption of the NFHMS nanofibers. Therefore, compared to NFMS and solid MS, the NFHMS provided the best control of the release of BFP from the microspheres (Figure 2e).

We first examined how NFHMS‐E7‐CaP/BFP regulated osteogenic gene expressions and enhanced osteogenesis. The in vitro data showed that the NFHMS‐E7‐CaP/BFP promoted BMSC differentiation and enhanced the expressions of osteogenic markers (ALP, RUNX2, SP7, and OCN) and calcium nodule formation. ALP is an early‐stage osteogenic differentiation marker and promotes mineralization by hydrolyzing pyrophosphate and generating inorganic phosphate.^[^ ^20^ ^]^ RUNX2 and SP7 are a transcription factors that regulate the expressions of osteogenic genes and promotes bone formation.^[^ ^21^ ^]^ The expressions of ALP, RUNX2 and SP7 in the NFHMS‐E7‐CaP/BFP were significantly higher than those in the other groups after incubation for 7 and 14 days. At day 14, the similar result was obtained for the expression of OCN that is a late osteogenic marker and plays a regulatory role in bone mineralization and calcium homeostasis.

To further investigate the effect of NFHMS‐E7‐CaP/BFP on periodontal bone formation, we developed a challenging rat fenestration defect model and implanted the NFHMS‐E7‐CaP/BFP in the defect site for up to 8 weeks. This fenestration defect model connects the defect to oral cavity and is challenging to heal using conventional GTR/GBR biomaterials. The μ‐CT images showed that the distance from CEJ to ABC in the NFHMS‐E7‐CaP/BFP group was the shortest after 8 weeks implantation. The decrease of the distance from CEJ to ABC is an important parameter for successful periodontal tissue regeneration. Most of the defect in the NFHMS‐E7‐CaP/BFP group were covered with newly formed bone, while only a small amount of new bone appeared in the defect area in the NFHMS and NFHMS‐E7 groups. Quantitative data indicated that the NFHMS‐E7‐CaP/BFP had the highest BV/TV and BMD among all five groups. We believe that the superior capability of the NFHMS‐E7‐CaP/BFP for alveolar bone regeneration lay in at least the two aspects. First, the E7 peptide conjugated on the surfaces of NFHMS selectively attached more BMSCs in the defect area. Second, the sustained release of BFP from the NFHMS‐E7‐CaP/BFP accelerated differentiation and biomineralization of the BMSCs, leading to fast alveolar bone formation. Meanwhile, the nanofibrous architecture of the NFHMS was favorable to osteogenesis.^[^ ^22^ ^]^ The immunohistochemical staining further showed that the expressions of ALP, SP7, RUNX2, DMP‐1, and OCN in the NFHMS‐E7‐CaP/BFP were much stronger compared to other groups, confirming that the multifunctional NFHMS‐E7‐CaP/BFP was a promising biomaterial in enhancing periodontal alveolar bone regeneration.

## 4 Conclusions

In this study, we designed and synthesized functionalized NFHMS‐E7‐CaP/BFP and evaluated its effectiveness for promoting alveolar bone regeneration in a challenging fenestration defect model. Several unique techniques were developed to fabricate the multifunctional NFHMS‐E7‐CaP/BFP. First, NFHMS were fabricated via integration of a double emulsification technique and a thermally induced phase separation process. Next, E7 peptide was conjugated onto the NFHMS via a surface coupling method. Furthermore, BFP peptide was entrapped in CaP NPs followed by encapsulating the BFP‐loaded CaP NPs into the NFHMS through a double emulsion process. The in vivo study showed that the NFHMS‐E7‐CaP/BFP significantly enhanced alveolar bone regeneration in a challenging fenestration defect model, indicating this functionalized NFHMS‐E7‐CaP/BFP is a promising injectable material for periodontal bone regeneration.

## 5 Experimental Section

### Materials

Gelatin (Type B, from bovine skin, 225 g Bloom), fluorescein isothiocyanate (FITC), 1‐ethyl‐3‐(3‐dimethylaminopropyl) carbodiimide hydrochloride (EDC), 2‐(N‐morpholino) ethanesulfonic acid (MES), N‐hydroxysuccinimide (NHS), glycine, calcium dichloride, and disodium hydrogen phosphite were purchased from Sigma‐Aldrich (St Louis, MO). Mineral oil, isopropanol, ethanol, and hexane were ordered from VWR Scientific (Seattle, WA). Bone forming peptide‐1 (BFP) and E7 peptide were synthesized by Biomatik Co. (Wilmington, DE). PrimeScript™ RT Reagent Kit and TB Green Premix Ex Taq II were ordered from Takara Bio (Mountain View, CA). Bone marrow derived stem cells (BMSCs) were purchased from Lonza (PT‐2501, Basel, Switzerland). Periodontal ligament cells (PDLSCs) were purchased from Celprogen (36085‐01, Torrance, CA). Fibroblasts (FBs) were purchased from ATCC (Pcs‐201‐018, Manassas, VA). Epithelial cells (EPCs) were purchased from Lifeline Cell Technology (FC‐0094, Walkersville, MD).

### Preparation of BFP‐Loaded Calcium Phosphate (CaP/BFP) Nanoparticles (NPs)

One milliliter of calcium chloride solution (400 mM) was mixed with 6 mg of BFP and was dispersed in cyclohexane to form microemulsion A. Next, one milliliter of disodium hydrogen phosphate solution (400 mM) was dispersed in cyclohexane to form microemulsion B, which was slowly added into the microemulsion A at a rate of 10 mL/hour with continuous stirring at room temperature. The resulting CaP/BFP NPs were centrifuged at 10 000 rpm for 15 min, washed with ethanol for 4 times, redispersed in double‐distilled water, and freeze‐dried. The CaP/BFP NPs were examined using scanning electron microscopy (SEM, JSM‐6010LA).

### Preparation of NFHMS, NFMS, and Solid MS

NFHMSs were fabricated by combining an oil/water/oil (O/W/O) double emulsification technique with a thermally induced phase separation process. First, gelatin was dissolved in water/ethanol (1/1, v/v) solvent mixture at 50 °C with different concentrations (6%, 12%, and 24%). Next, mineral oil (3 mL, 7 mL, or 10 mL) was gradually added into 10 mL of gelatin solution under rigorous mechanical stirring to form an oil‐in‐water emulsion. Mineral oil (50 mL) was gradually added into the emulsion to form an oil‐in‐water‐in‐oil double emulsion and stirred for different durations (30 sec, 3 min, 8 min, and 15 min). Subsequently, the mixture was poured into 800 mL of isopropanol/hexane/ethanol (4/3/1, v/v/v, −80 °C) mixture under gentle stirring to induce phase separation and solvent exchange. The obtained NFHMSs were dispersed in acetone/water solvent (9/1, v/v) and crosslinked using EDC and NHS in a MES buffer (pH 5.3, 0.05 M) at 4 °C for 24 h. The NFHMSs were washed with 100% ethanol and 50% ethanol for10 min, separately, and incubated in a glycine solution for 3 h to neutralize the unreacted crosslinking agent. The NFHMS were washed sequentially with 50%, 70%, and 100% ethanol. Four mesh sieves (mesh sizes of 150 µm, 90 µm, 45 µm, and 32 µm) were stacked and used to separate the NFHMS. The NFHMS suspension was first poured into the 150 µm mesh sieve and shook for 5 min to remove macroaggregates and large microspheres (>150 µm). Next, the NFHMS with size of 150–90 µm, 90–45 µm, and 45–32 µm were separated by sequentially using the mesh sieves with the sizes of 90 µm, 45 µm, and 32 µm. The obtained NFHMSs were lyophilized and stored for later use. The surface morphology and hollow structure of the NFHMSs were observed using SEM and confocal microscopy (Leica STP6000). For comparison, nanofibrous microspheres without hollow structure (NFMS) were fabricated by combining a water/oil (W/O) emulsification technique and a thermally induced phase separation process. Except that no mineral oil was added to the gelatin emulsion, all other steps were the same to those of the NFHMS preparation. Solid MS that have solid walls and smooth surfaces were prepared using a conventional solvent evaporation technique as described in our previous study.^[^ ^23^ ^]^ The degradation of the microspheres (NFHMS, NFMS, and solid MS) was tested by measuring the weight loss of the microspheres at different time points. Briefly, 10 mg of NFHMS, NFMS, and solid MS was immersed into 1 mL PBS and incubated for 1, 2, 5, 8, 13, 18, 23, 29, and 35 days. At each time point, the microspheres were freeze‐dried, and the weights were measured using a precision balance. The elastic modulus and hardness tested by nanoindentation were used to characterize the strength performance of NFHMS. Nanoindentation analysis was conducted by using a nano‐test apparatus (Hysitron TI‐PREMIER).

### Preparation of E7‐Modified CaP/BFP‐Loaded NFHMSs (NFHMS‐E7‐CaP/BFP)

To encapsulate CaP/BFP NPs into NFHMSs (NFHMS‐CaP/BFP), the CaP/BFP NPs were dispersed into the gelatin solution at the first step of emulsion, and the other procedure was the same to that of NFHMS preparation. NFHMS‐E7‐CaP/BFP was prepared by conjugating E7 peptide onto the surface of NFHMS‐CaP/BFP. Briefly, NFHMS‐CaP/BFP (60 mg, 1.2 µmol) was dispensed in water, and sulfo‐smcc (5.2 mg, 12 µmol) was added to the NFHMS‐CaP/BFP and stirred for 30 min at room temperature. Next, E7 peptide (14 mg, 12 µmol) was added dropwise and was continuously stirred for 8 h. The acquired NFHMS‐E7‐CaP/BFP was washed and lyophilized. The NFHMS‐CaP/BFP and NFHMS‐E7‐CaP/BFP were imaged under confocal microscopy to observe the encapsulation of CaP/BFP into NFHMS and the conjugation of E7 onto the surfaces of NFHMS‐CaP/BFP. For comparison with the NFHMS‐E7‐CaP/BFP, E7 was also conjugated onto the surfaces of NFMS‐CaP/BFP and solid MS‐CaP/BFP, separately. The E7 peptide content in NFHMS‐E7 was measured using fluorescence plate reader (Biotek Cytation 5) based on the emission wavelength of tryptophan residue (W) at 350 nm upon excitement at a 280 nm wavelength.

### In Vitro Release of BFP

To determine the release profiles of BFP from the microspheres, the NFHMS‐E7‐CaP/BFP, NFMS‐E7‐CaP/BFP, and Solid MS‐E7‐CaP/BFP were immersed in 1 mL of PBS and stirred at 37 °C. At designated time points (1, 2, 3, 6, 9, 12, 16, and 21 days), the supernatant was collected and replaced with the same amount of fresh PBS. After the last collection, the samples were sonicated and the BFP in the supernatant was defined as the unreleased BFP in the microspheres. All the samples were analyzed by UV‐Vis.

### Cell Attachment and Proliferation on Microspheres

The microspheres (NFHMS, NFHMS‐CaP/BFP, NFHMS‐E7, and NFHMS‐E7‐CaP/BFP) were soaked in 75% ethanol for 30 min and washed three times with PBS. Next, the microspheres were immersed in α‐MEM with 10% FBS and pipetted into sterile 24‐well plates. Bone morrow derived stem cells (BMSCs), periodontal ligament cells (PDLSCs), gingival fibroblast cells (FBs), and gingival epithelial cells (EPCs) were seeded onto the microspheres, separately, and cultured for 1 h, 4 h, or 24 h. The constructs were fixed with 4% paraformaldehyde and incubated with phalloidin 633 for 45 min. After washed with PBS, the samples were incubated with Hoechst to stain the nucleus. At 24 h, the samples were fixed, dehydrated by a series of graded ethanol solutions, and analyzed using SEM.

To further measure the selective adhesion to microspheres, the same numbers of BMSCs, PDLSCs, FBs and EPCs were blended and seeded onto the microspheres. The BMSCs, PDLSCs, FBs and EPCs were stained with LysoTracker™ Deep Red, Calcein‐AM, CellTracker™ Orange CMTMR Dye, and Hoechst 33342, respectively. The cells on the microspheres were co‐cultured for 24 h. The proportion of each type of cells on the microspheres was determined using flow cytometry. In addition, the viabilities of BMSCs on the NFHMS, NFHMS‐CaP/BFP, NFHMS‐E7 and NFHMS‐E7‐CaP/BFP were assessed by MTT assay at day 1, 3 and 5 after cell seeding. The MTT test was performed according to manufacturer's instructions.

### Alkaline Phosphatase (ALP) Activity and Alizarin Red (ARS) Staining

The cell‐seeded NFHMS, NFHMS‐CaP/BFP, NFHMS‐E7, and NFHMS‐E7‐CaP/BFP were cultured in an osteogenic medium and examined for ALP staining at day 7. Each group was fixed with 4% paraformaldehyde and changed to ALP substrate solution (5 µL NBT and 3.75 µL BCIP into 1 mL ALP buffer). Twenty minutes later, the reaction was stopped by washing with distilled water. For ARS staining, each group was cultured in an osteogenic medium for 14 days, fixed with 4% paraformaldehyde, stained with ARS solution (pH 4.2) for 30 min, and washed with distilled water. Both ALP and ARS staining were photographed with an Olympus SZX16 stereomicroscope. To quantify the amount of ARS bound to the mineralized nodules, the bound ARS was dissolved in 10% cetylpyridinium chloride (CPC), and the OD values at 570 nm were measured using a Biotek cytation 5 microplate reader.

### In Vitro Gene Expression

RT‐PCR was performed to evaluate gene expressions on the NFHMS, NFHMS‐CaP/BFP, NFHMS‐E7, and NFHMS‐E7‐CaP/BFP. After cultured in an osteogenic medium for 14 days, total RNA was isolated using a RNeasy Mini Kit (Qiagen) according to the manufacturer's protocol and then transcribed into cDNA using PrimeScript™ RT Reagent Kit (Takara). The resulting cDNAs were used as templates for quantitative RT‐PCR (qRT‐PCR) using TB Green Premix Ex Taq II (Takara), and performed using Bio‐Red CFX96TM (BioRad, USA). GAPDH was used as an internal normalizing control for mRNA. Relative quantification of gene expression was analyzed using 2^−ΔΔ^CT method. The primer sequences are listed in Table S1 (Supporting Information).

### In Vivo Surgical Procedure

All animal protocols were approved by the University Committee on Use and Care of Animals of Texas A&M University School of Dentistry (Protocol # IACUC 2020‐0284‐CD). A total of 40 Sprague Dawley (SD) rats (Charles River, 180–200 g) were randomly assigned to five groups (*n* = 8): control, NFHMS, NFHMS‐CaP/BFP, NFHMS‐E7 and NFHMS‐E7‐CaP/BFP groups. The rats were anesthetized with isoflurane and followed by an intraperitoneal injection of ketamine/xylazine. After removed the furs around the surgical area, iodine disinfection was performed on the surgical site, and a 15 mm incision was performed to expose in an anterior‐posterior direction the surface of the masseter muscle. Next, a second incision was used to dissect through the masseter muscle slightly under the lower ligamentous line until it reached the body of the mandible (buccal plate). A No. 4 high‐speed round bur was used to initiate access to the buccal root, followed by a No. 1/2 high‐speed bur to properly remove cementum on both side of the root and create a rat mandibular periodontal intrabony defect (3 mm x 10 mm x 1 mm). Sterilized microspheres (NFHMS, NFHMS‐CaP/BFP, NFHMS‐E7, and NFHMS‐E7‐CaP/BFP) were implanted into the defect area. After that, the masseter muscle over the surgical site was repositioned using absorbable sutures, followed by suturing the skin incision to avoid the microspheres potentially moving out of the defect area. After surgery, all the rats were fed with a soft‐food diet for 7 days. The samples were harvested at 4 and 8 weeks after surgery.

### µ‐CT Analysis, Histological, and Immunohistochemical Examination

Samples were analyzed using a µ‐CT35 imaging system (Scanco Medical, Basserdorf, Switzerland). The bone density of bone volume to total volume ratio (BV/TV) and bone mineral density (BMD) were obtained using a Scanco software. The distance between the cemento‐enamel junction (CEJ) and alveolar bone crest (ABC) was measured using ImageJ. Samples were fixed, demineralized, dehydrated, and subsequently embedded in paraffin. The sections with a thickness of 5 µm were processed for histologic observation using Hematoxylin and Eosin (H&E) staining and Masson's trichrome staining. For immunohistochemical analysis, samples were de‐paraffinized and reacted with ALP, specificity protein‐7 (SP7), runt‐related transcription factor 2 (RUNX2), dentin matrix acidic phosphoprotein 1 (DMP‐1) and osteocalcin (OCN). All the immunohistochemistry experiments were detected with Vectastain Elite ABC‐HRP Kit (Vector Laboratories, Burlingame, CA) according to the manufacturer's instructions.

### Statistical Analysis

Data were analyzed with SPSS software (version 19.0). All results were expressed as mean ± standard deviation. The differences among the groups were compared by one‐way ANOVA. To test the significance of the observed differences between two groups, an unpaired Student's t‐test was applied. The significance level was set at *p* < 0.05.

## Conflict of Interest

The authors declare no conflict of interest.

## Author Contributions

Q.L. performed conceptualization, methodology, validation, investigation, data analysis, and writing‐original draft. C.M. performed methodology. Y.J. performed data analysis. X.L. performed conceptualization, data analysis, writing‐review & editing, and supervision.

## Supporting information

Supporting Information

## Data Availability

The data that support the findings of this study are available from the corresponding author upon reasonable request.
