# Supplementary material for: Multifunctional Nanofibrous Hollow Microspheres for Enhanced Periodontal Bone Regeneration
Source: Adv Sci (Weinh). 2024 May 17;11(28):2402335. doi: 10.1002/advs.202402335 (PMC11267322; doi:10.1002/advs.202402335)
Supplement: Supplementary file 1 — Supporting Information [file ADVS-11-2402335-s001.docx]

**Supporting Information**

**Multifunctional Nanofibrous Hollow Microspheres for Enhanced Periodontal Bone Regeneration**

Qian Li,^1,2^ Chi Ma,^3,4^ Yan Jing,^5^ and Xiaohua Liu^1, 2^*

*^1^Department of Biomedical Sciences, Texas A&M University School of Dentistry, Dallas, TX 75246*

*^2^Chemical and Biomedical Engineering Department, University of Missouri, Columbia, MO 65211*

*^3^Center of Excellence in Hip, Scottish Rite for Children, Dallas, TX, 75219*

*^4^Department of Orthopedic Surgery, University of Texas Southwestern Medical Center, Dallas, TX 75390*

*^5^Department of Orthodontics, Texas A&M University School of Dentistry, Dallas, TX 75246*

*Correspondence to:

Xiaohua Liu, PhD

Professor

*Chemical and Biomedical Engineering Department*

*University of Missouri, Columbia, MO 65211*

*Phone: 573-882-6497*

*Email:* [*xlz2y@missouri.edu*](mailto:xlz2y@missouri.edu); *xliu1@tamu.edu*

Table S1. Primer sequences for qRT-PCR.

| Gene | Primer sequence |
| --- | --- |
| ALP | F: ACTGCGCTCCTTAGGGCT  R: GGCAGCGTCAGATGTTAATTG |
| RUNX2  SP7 | F: AGTGCTCTAACCACAGTCCATGCA  R: TACAAACCATACCCAAGTACCTGTTT  F: GCCAGTAATCTTCGTGCCAG  R: AGTGAGCTTCTTCCTGGGGA |
| OCN | F: GGTAGTGAACAGACTCCGGC  R: CAAGCAGGGTTAAGCTCACA |
| GAPDH | F: TTGATGGCAACAATCTCCAC  R: CGTCCCGTAGACAAAATGGT |

Table S2. Comparison of structural parameters among NFHMS with different processing parameters.

| Microspheres | Porosity (%) | Apparent density (g/dm^3^) | Fiber diameter (nm) | Fiber length (nm) |
| --- | --- | --- | --- | --- |
| 6% NFHMS | 95.86±0.27 | 43.34±1.37 | 250±87 | 1749±507 |
| 12% NFHMS | 92.74±0.46 | 76.01±4.49 | 295±85 | 1367±290 |
| 24% NFHMS | 84.18±2.04 | 165.23±14.86 | 480125 | 462±114 |
| 12% NFMS | 87.92±0.93 | 126.55±11.07 | 271±94 | 1342±263 |
| solid MS | 1.63±0.18 | 1033.01±89.68 | - | - |

Table S3. Thickness of the NFHMS with different volume of inner oil.

| Volume of inner oil | 3 ml | 7 ml | 10 ml |
| --- | --- | --- | --- |
| Thickness (μm) | 18.9±1.4 | 11.3±0.6 | 2.6±0.4 |


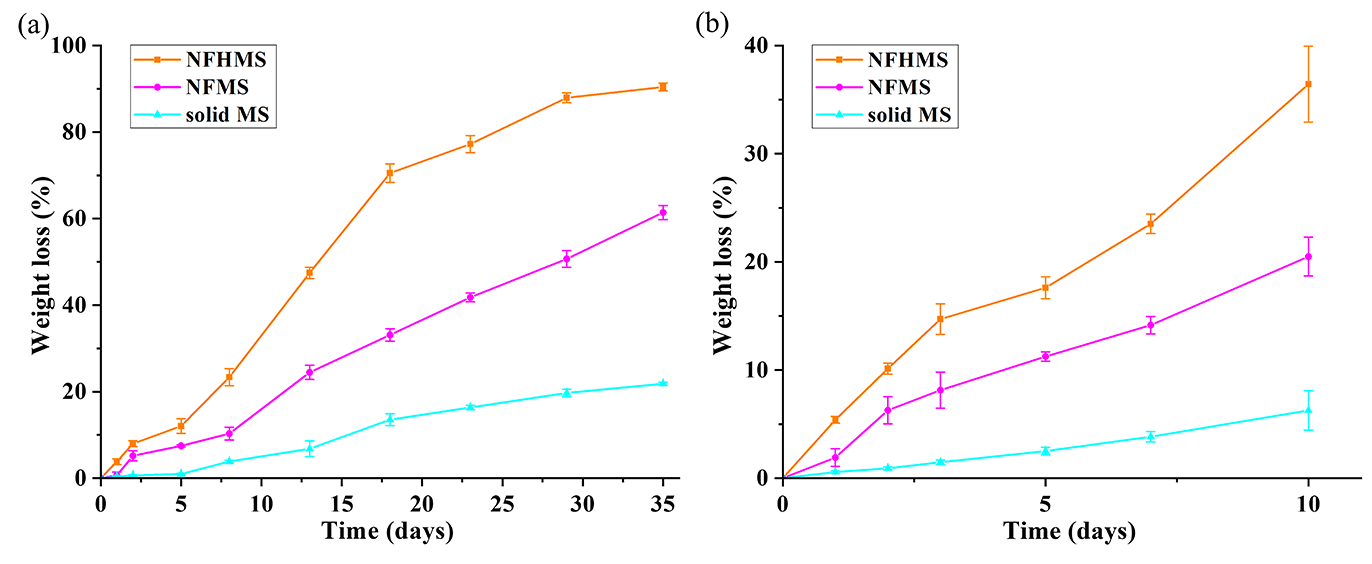


Figure S1. Degradation rate of NFHMS, NFMS and solid MS.


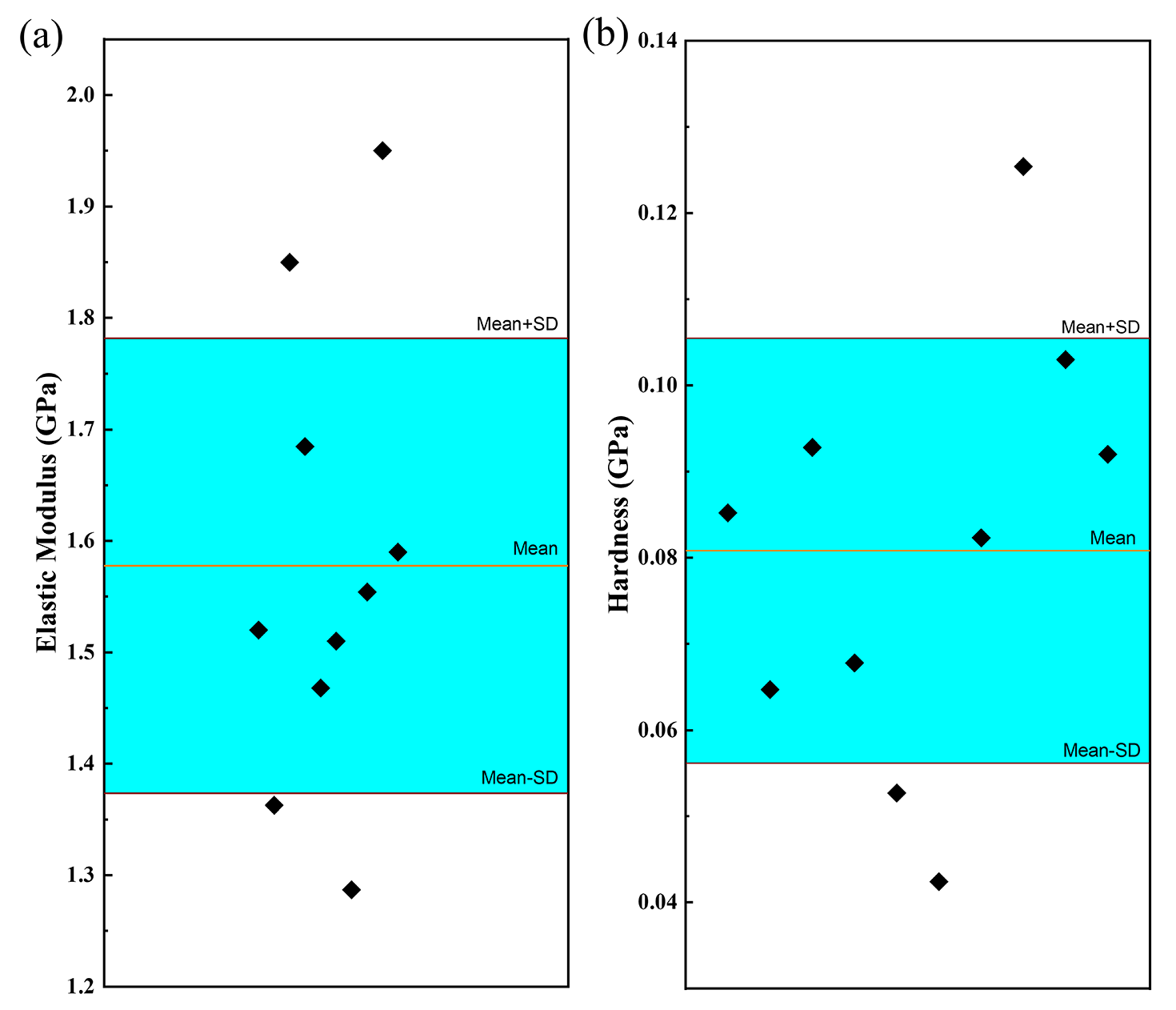


Figure S2. Elastic modulus data (a) and hardness (b) of NFHMS.


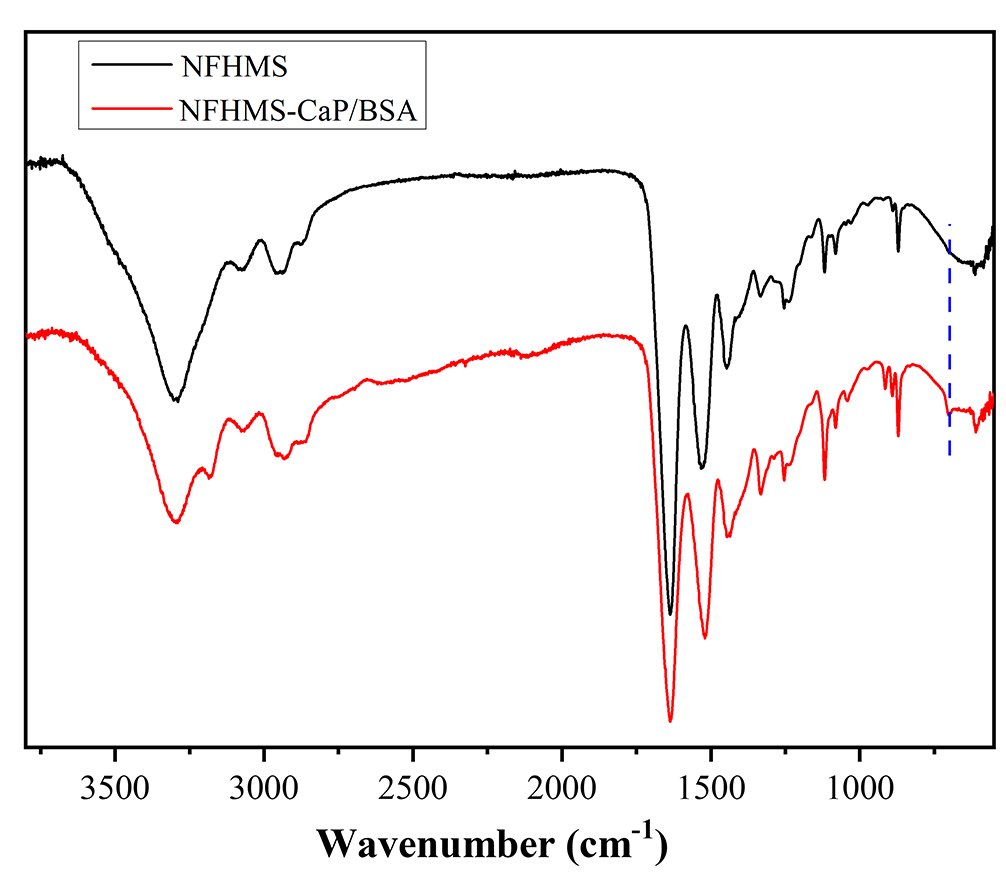


Figure S3. FT-IR spectra of NFHMS and NFHMS-CaP.


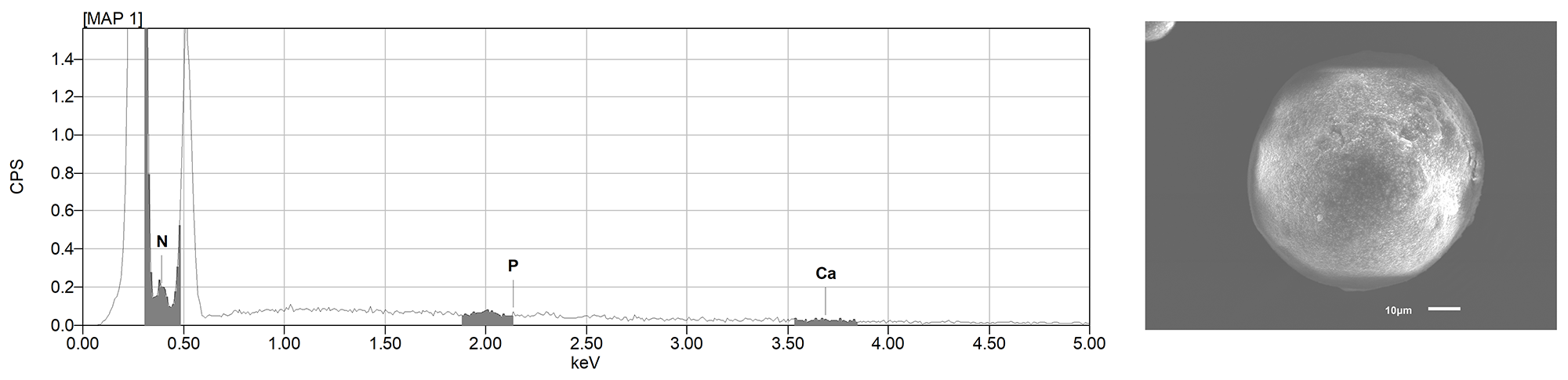


| Chemical fomula | Mass% | Atom% | Sigma | Net | K ratio | Line |
| --- | --- | --- | --- | --- | --- | --- |
| N | 58.70 | 78.18 | 2.30 | 712 | 0.0022678 | k |
| P | 18.91 | 11.39 | 0.86 | 1449 | 0.0020675 | k |
| Ca | 22.39 | 10.42 | 1.44 | 823 | 0.0031920 | k |
| Total | 100.00 | 100.00 |  |  |  |  |

Figure S4. EDS spectra and elemental analysis data for NFHMS-CaP/BSA.


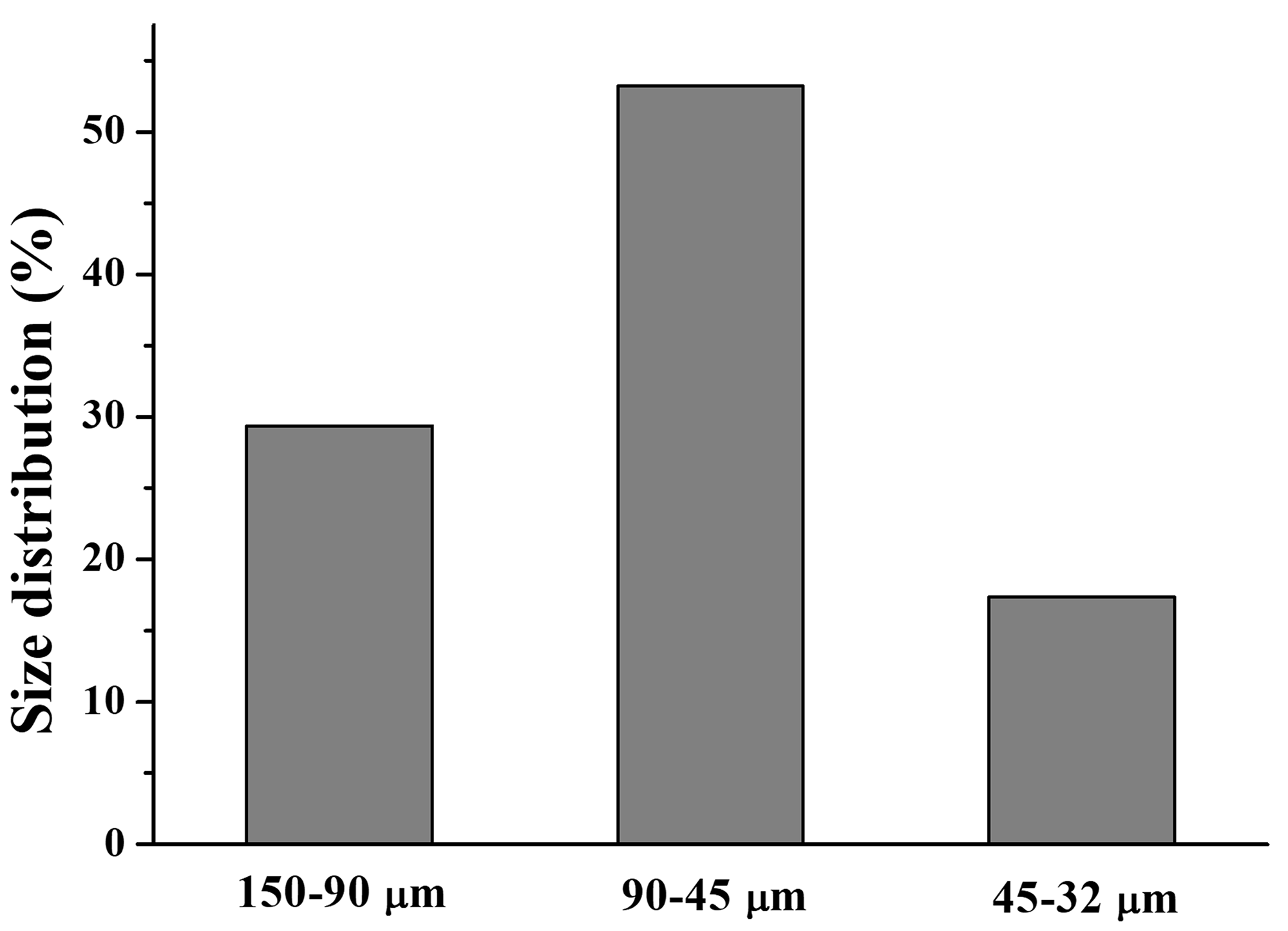


Figure S5. Size distributation of NFHMS-E7-CaP/BFP.


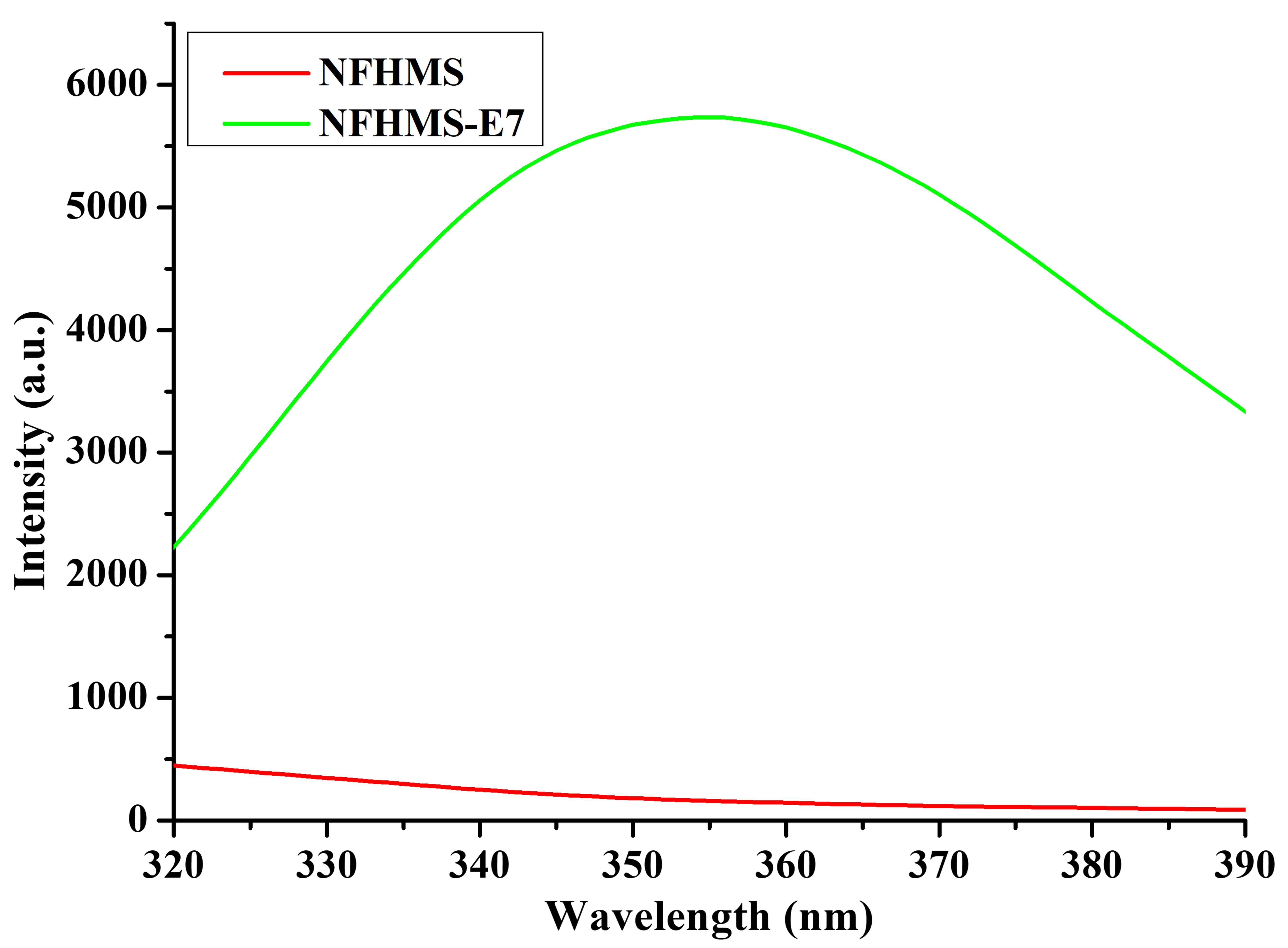


Figure S6. Fluorescence emission spectra of NFHMS-E7.


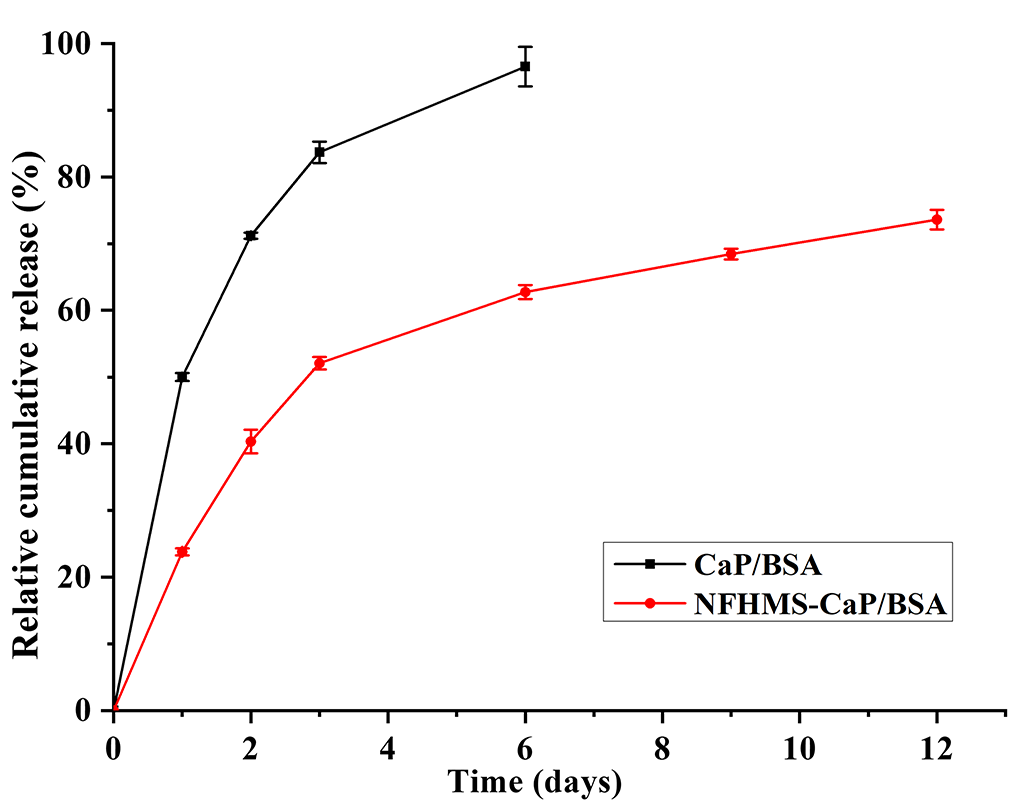


Figure S7. Release profiles of BSA from CaP/BSA and NFHMS-CaP/BSA. N=3.


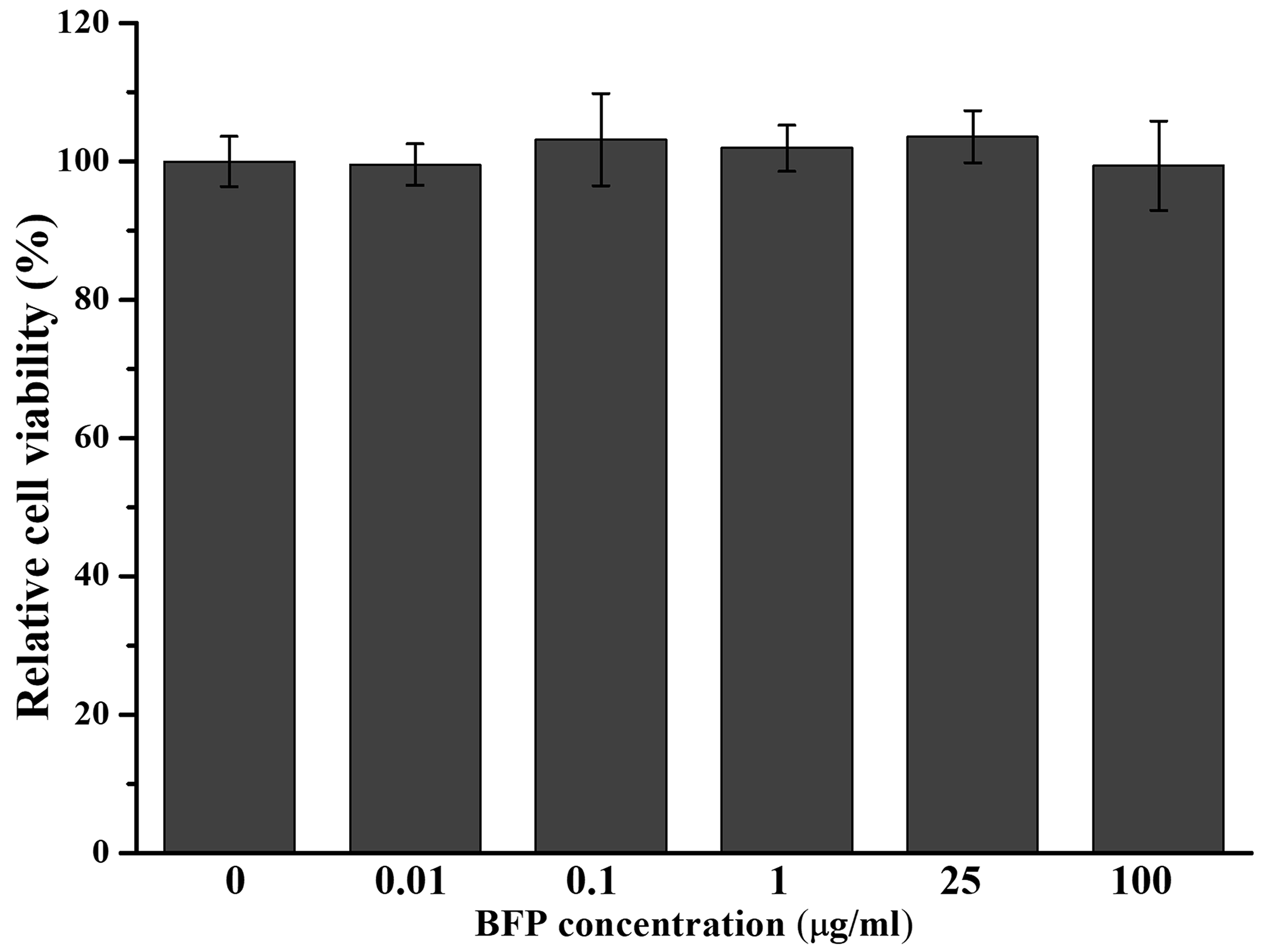


Figure S8. Relative cell viability of BMSCs at different BFP concentration after treated for three days. BFP concentration ranging from 0.01 to 100 μg/ml.


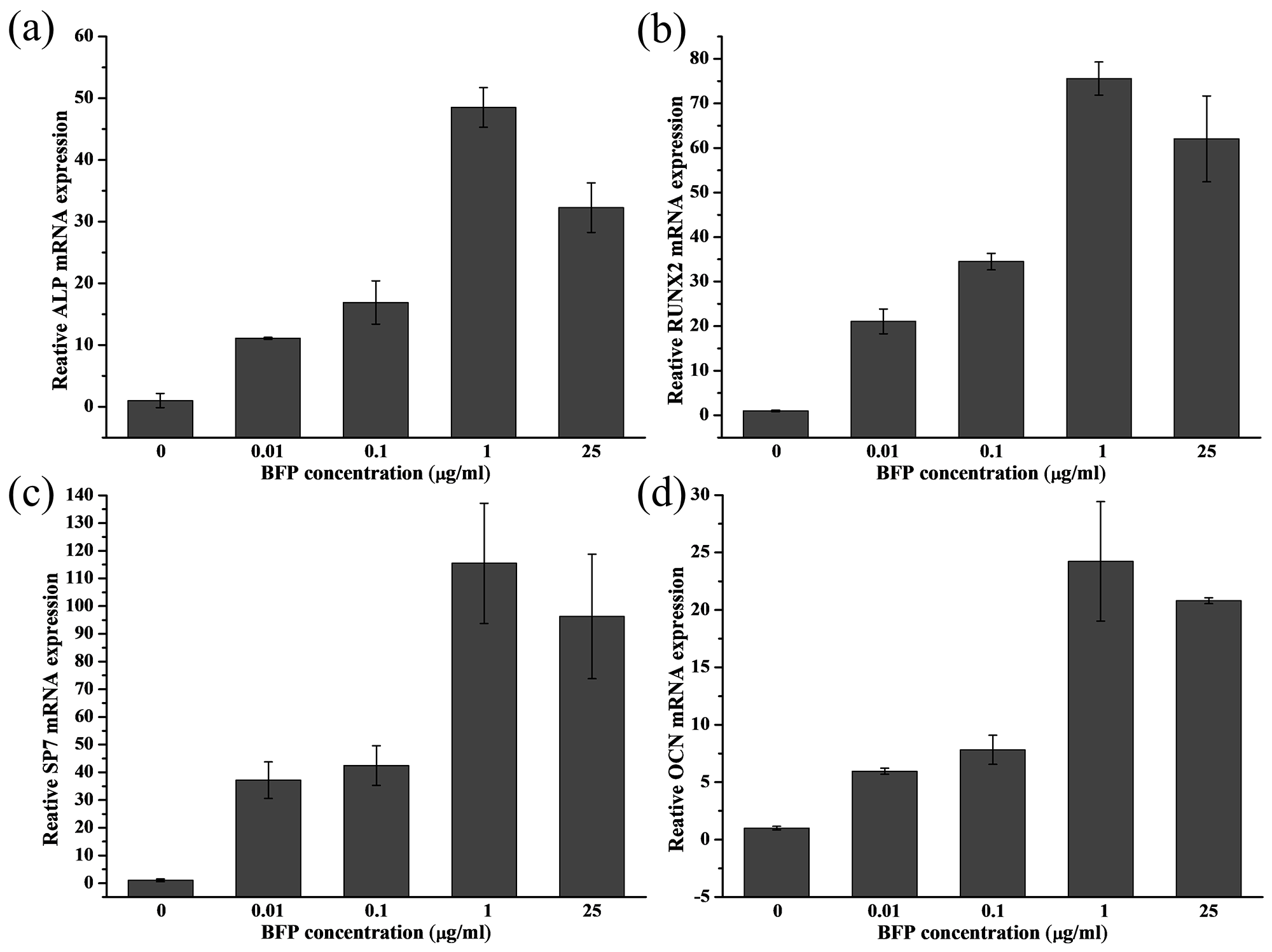


Figure S9. Osteogenic differentiation of BMSCs at different BFP concentration for 14 days. (a) ALP expression, (b) RUNX2 expression, (c) SP7 expression, and (d) OCN expression. N=3.
